# A Presence-Based Context-Aware Chronic Stress Recognition System

**DOI:** 10.3390/s121115888

**Published:** 2012-11-16

**Authors:** Klemen Peternel, Matevž Pogačnik, Rudi Tavčar, Andrej Kos

**Affiliations:** 1 Laboratory for Telecommunications, Faculty of Electrical Engineering, University of Ljubljana, Tržaška 25, 1000 Ljubljana, Slovenia; E-Mails: matevz.pogacnik@fe.uni-lj.si (M.P.); andrej.kos@fe.uni-lj.si (A.K.); 2 Mirabi Institute, Hrastje 223, 4000 Kranj, Slovenia; E-Mail: rudi.tavcar@amis.net

**Keywords:** presence, chronic stress, context awareness, embedded sensors, Hidden Markov models, health monitoring

## Abstract

Stressors encountered in daily life may play an important role in personal well-being. Chronic stress can have a serious long-term impact on our physical as well as our psychological health, due to ongoing increased levels of the chemicals released in the ‘fight or flight’ response. The currently available stress assessment methods are usually not suitable for daily chronic stress measurement. The paper presents a context-aware chronic stress recognition system that addresses this problem. The proposed system obtains contextual data from various mobile sensors and other external sources in order to calculate the impact of ongoing stress. By identifying and visualizing ongoing stress situations of an individual user, he/she is able to modify his/her behavior in order to successfully avoid them. Clinical evaluation of the proposed methodology has been made in parallel by using electrodermal activity sensor. To the best of our knowledge, the system presented herein is the first one that enables recognition of chronic stress situations on the basis of user context.

## 1. Introduction

Stress is a physiological, psychological and behavioral response to every change people must adapt to. The stress reaction was first described by Selye [1]. A healthy organism returns to its normal state after a reaction to acute stress. Unfortunately, in the modern world, many stressors are not acute and often reoccur.

Ongoing chronic stress has a long-term negative effect on the physical as well as the mental health of an individual [2,3]. It contributes to a range of diseases such as elevated arterial pressure, arrhythmia, acute myocardial infarction, cerebrovascular insult, diabetes, osteoporosis and hyperlipidemia (increased levels of fats in the blood). The psychological effect of chronic stress is mostly linked to the onset of depression, but it also affects our cognitive functions (perception and logical thinking) and can intensify emotional responses which affect interpersonal relationships. One of the negative consequences is also reduced work efficiency.

Chronic stress is difficult to tackle, because the problem starts with the detection of stressors through the measurement of related parameters. Measurements are currently done through interviews and questionnaires. The problem with this method is the fact that the results usually don't offer a complete picture, because the patient often can't recall the entire history of encounters with different stressors. In most cases we are able to capture the current snapshot, but not the entire past situation. The questionnaires and interviews mostly act as a necessary input of a personalized interpretation of the measurements, which we obtain using different methods.

For a thorough and more reliable identification of chronic stress the measurements need to be conducted continuously, throughout a longer time period. This can be done with the use of different electronic measurement devices that periodically, based on physiological indicators, can evaluate the current level of stress. Such indicators (markers) include: electrodermal activity (EDA), various pulse samples, blood pressure and respiration activity. On today's market, we can already find small sensor devices that allow these types of measurements.

However, even this approach, has its drawbacks. These are mostly related to the fact that the measurements are not taken in a controlled environment, but during the individual's everyday activities. Consequently the signals acquired by sensors are often influenced by the patient's movements during the measurement (motion artifact) [4]. Results can also be distorted by the users' physical activity during or shortly before the measurement is taken. Elevated heart rate for example caused by physical activity can cause erroneous stress detection.

An alternative option of measuring an individual's exposure to different stressors is the use of different self-reporting methods [5–7]. They presuppose the users' reporting on the exposure to stress at predefined time intervals, at predetermined events or when they receive signals from various devices (signal-contingent recording).

Measuring chronic stress with the use of the signal-contingent recording method is usually done with the help of a beeper or a wristwatch, which triggers a sound alarm at a random time during the day. The method is especially appropriate for recording emotional states (mood) that change during the day and are difficult to recall at a later time, and for reoccurring events.

One weakness of the method is the amount of cooperation it requires from the individual. At times, the reporting moment can be inconvenient (e.g., at dinner, while driving, during a meeting, in the shower), which results in missing measurement data.

In this paper we propose an architecture which combines the signal-contingent recording method with the usage of a presence based context recognition system. We present a solution, which enables the measurement of chronic stress solely on the users' context that can be calculated inside extended presence platform. With the use of an appropriate graphical representation of potential stressors to the user, these can be expeditiously identified and acted on accordingly. In case an occurring stressful event is identified (e.g., traveling in the morning rush hour), the user can alleviate or even eliminate the stressor by consciously applying different solutions such as using public transportation or driving to work thirty minutes earlier, for example.

The main contribution of presented work is the definition of comprehensive system architecture, selection of appropriate data sources and proposal of dynamic system model which enables us to simplify training and maintain a good relationship between high usability and expected results.

The remainder of the paper is organized as follows: in Section 2 related work is presented. In Section 3 user context and existing standardized presence architecture overview is given. Section 4 covers conceptual and technical details regarding context-aware stress recognition system, and Section 5 deals with the results of the proposed methodology. In Section 6 a clinical evaluation is represented and in Section 7 conclusions are given and future work is predicted.

## 2. Related Work

One of the most common methods of recognizing user's current emotional experience includes video data analysis. Here we are referring to techniques which deal with the process of recognizing human facial expressions based on selected features. According to Hoque, McDuff and Picard [8] they can provide accuracy of 92% distinguishing smiling reactions on frustrated and delighted stimuli using a dynamic SVM classifier. Anisetti and Bellandi [9] further use Russel-based classification in order to classify emotional state of the subject based on face related features.

In recent research, the usage of various body sensors is emphasized when trying estimating stress. As an example, EDA is one of the most robust physiological indices of stress [10] with many measuring devices available on the market. Some researchers [11,12] also suggest that heart rate variability parameter can be used to separate the activation level of sympathetic (SNS) and parasympathetic (PNS) nervous system while others use a larger number of physiological features to determine the existence of the stress response [13]. Some very interesting experiments have been conducted in the real world. Healey and Picard [14] present methods for collecting and analyzing multiple physiological data (ECG, electromyogram, EDA and respiration) during driving tasks to determine a driver's relative stress level. They collected data of at least 50-min duration from 24 drivers. Features were extracted from 5-min intervals of data during the rest, highway and city driving conditions to distinguish three levels of driver stress with an accuracy of over 97% across multiple drivers and driving days. Results also show that for most drivers skin conductivity (EDA) and heart rate variability provides the highest correlation to stress level.

When asked about current social and technology trends, one cannot help to think about the growing number of multitude smart consumer devices that surround us. Functionality originally reserved for personal computers is today being offered on modern mobile phones and tablet devices. In addition, a number of technologies have matured in the past years, such as numerous wireless and embedded sensor technologies, which are finding their way into modern consumer devices. With the democratization of the aforementioned technologies, researchers try to create new automated methods in order to estimate user's stress. Bauer and Lukowicz [15] have recently described some initial results from an ongoing project. They use mobile phone sensors to detect stress related situations based on location traces, Bluetooth devices seen during the day and phone call patterns. The results show that a behaviour modification can clearly be seen, but the exact interpretation and generalization still requires further work.

Taken together, the concepts and approaches presented here are diverse and mostly serve very well in controlled environments or for conducting short-term measurements (e.g., approaches like video analysis). For a reliable assessment of chronic stress the measurements need to be done continuously by using robust sensor technologies (to avoid motional artifacts). In order to get the best long-term results and maximize usability we can find various existing sensor technologies inside user devices the most appropriate to obtain various stress related data.

## 3. Presence Based Context-Aware Architecture

User context is mostly described by one or a group of data parameters, which deliver information about a state of a user (e.g., location, activity, identity, time) at a certain point in time. A more general definition of the term ‘context’, defines it as “any information that can be used to assess the situation the chosen entity is in” (e.g., the user) [16]. In a time when we are surrounded by a multitude of sensor equipped devices that are connected to the network, there are new possibilities to obtain a variety of information about the users' activity. The latter can give us an accurate image of users' context and acts as enabler for building new services (Context-aware Services—CaS). Such services may be much more “intelligent” and bring new value to the users.

For a precise determination of the context, it is of course necessary to define a broad range of different data sources that can be arbitrarily numerous and cover virtually all dimensions of user's behavior, communication habits and social interactions within the selected community [17]. Nowadays, a number of researchers struggle with the development of new holistic approaches [18] based on combination of such data fragments.

**Figure 1.**
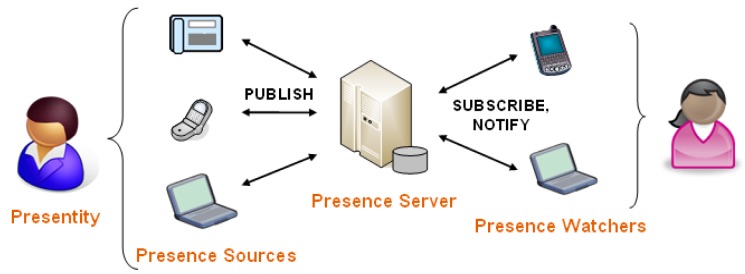
Basic architecture of a presence system specified by OMA.

An example in the field of information and communication technologies is a system that can detect user context and consequently reason about its state of accessibility at any moment [19]. Such a service is called a presence service. It can recognize various situations, e.g., where the user is seated in the conference room. The state of accessibility is thus marked as “busy”, which means that every incoming call to his/her mobile telephone will be automatically forwarded to voicemail or even rejected. General architecture of the presence service is specified by the Open Mobile Alliance (OMA) [20] and comprises three basic components [21,22] presented in Figure 1:

- **Presence Sources** collect presence information and send it to the presence server. Presence information usually relates to a human, group of people, a computer program, or a similar entity. Each such entity that chooses to appear as a single actor to the presence service, is called a principal. Each principal can be assigned a conceptual model—presentity, encompassing and representing all of its presence information. Information on a presentity is collected by presence user agent elements (presence sources). Their function is to make presence information available to the presence server in a standardized data format and over a standardized interface.
- **Presence Server** receives and manages published presence information and allows watchers to request it or subscribe to it. Presence server is the central element of OMA presence architecture, gathering presence information from different presence agents, maintaining a database of presence information about individual presentities and finally serving composed information to watchers that request it. Presence server also executes authorization rules over watchers, composition and filtering of unwanted presence information.
- **Presence Watchers** obtain presence information from the presence server and consume it. Generally there are two types of watchers: Fetchers requesting the presence information when they need it, and Subscribers, which receive notification on any change of presence information.

Presence server acts as a centralized context-aware data aggregator and enables various external sources (e.g., sensors) to commit their data. While presence service architecture is already well defined, we can use it in order to define our own context-aware system which will perform stress calculation tasks. The latter can be generally divided into capturing of data from the environment (by the use of different sensors), gathering, abstraction and comprehension of the captured data (connection with the user context) and modification of the stress visualization application (watcher) in reference to the assessed context of the user.

According to Dey [16] we can define certain types of context-related information that is more important than others and provide insight into the current context. We recognized it as user's location (LOC), activity (ACT) and ambience (AMB). Within platform for chronic stress assessment we use two different presence data sources that provide information on the user (principal) context (Table 1):

- smart mobile phone and
- personal computer (PC).

Smart phones gather a number of information about the user and are at the same time aware of their surrounding through the use of different sensors. One of the fastest growing smartphone platforms is the Android platform. Being an open-source platform makes it especially interesting for the development of different applications in the field of user context awareness.

**Figure 2.**
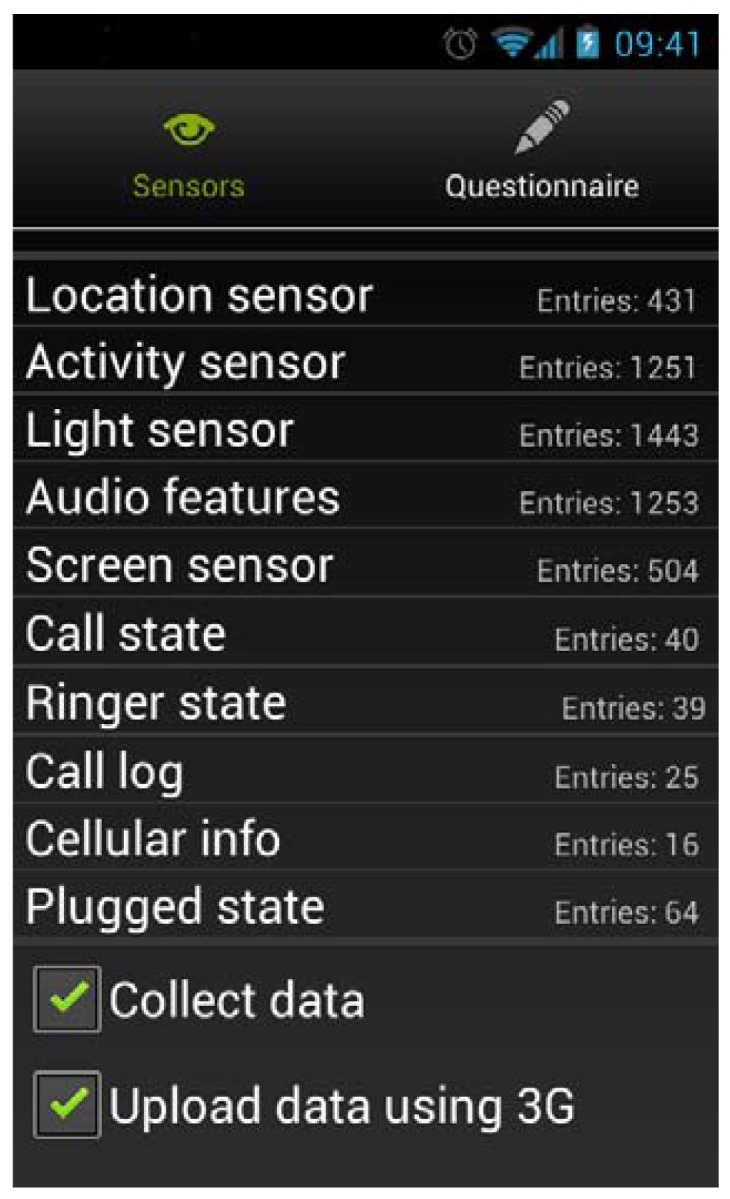
Mobile application for collecting user data.

A sensing framework named Fünf [23] was developed by the MIT Media Lab, and offers the possibility of fast mobile applications development for the capture of a multitude of contextual data inside Android. Based on an extended framework (we added some additional probes), we developed an application that captures and sends different user data to the server (Figure 2).

**Figure 3.**
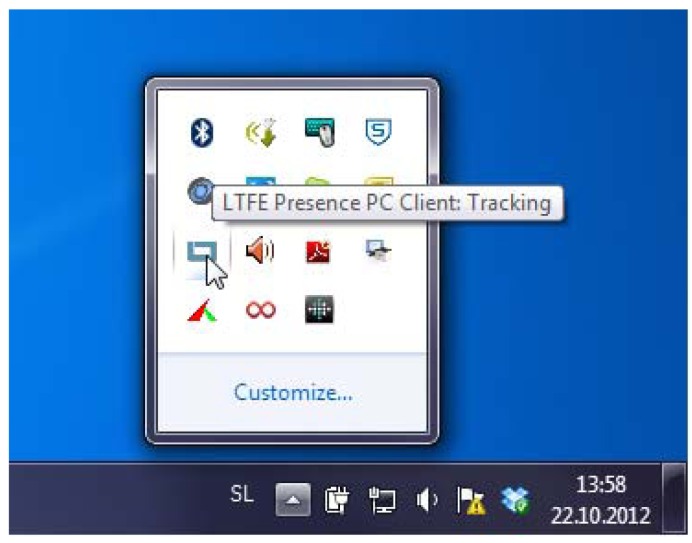
Windows background application for collecting PC-activity data.

Another application (Figure 3) was developed for the personal computer that functions in the background and senses keystrokes on a keyboard (keystroke logging) and the mouse movement in order to collect performance data [24].

Context-aware stress recognition system presents nonintrusive alternative of measuring chronic stress. The solution is a part of broader presence system and consists of:

- main context-aware platform
- data sources and
- chronic stress visualizer.

All components are described in further section. Also the overview of the implemented algorithm is given and a part of sensor data training set is presented.

## 4. Context-Aware Stress Recognition System

### 4.1. Platform Description

**Figure 4.**
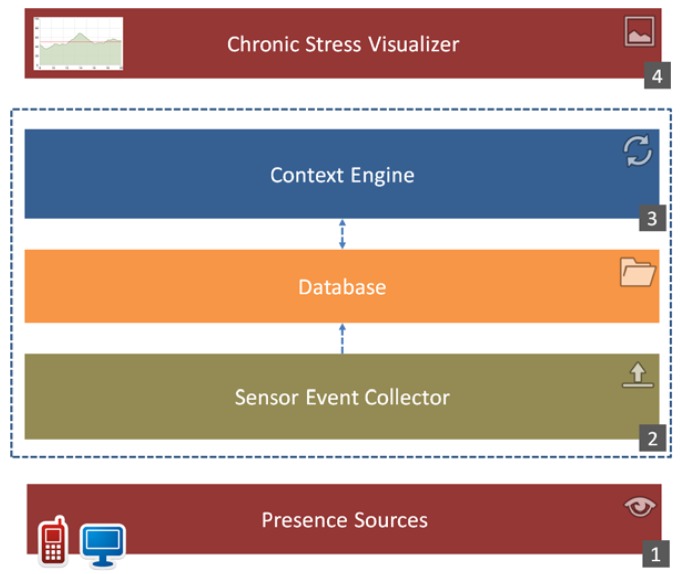
Context-aware Chronic Stress Recognition System (CaCSRS) architecture.

Our solution was integrated into the Internet of Things (IoT) platform named Occapi and consists of four main building blocks, presented in Figure 4: Presence Sources (1) that capture real life information, the Sensor Event Collector (2) that receives and filters all captured data sent by sources, the Context Engine (3) that provides current stress evaluation based on user context and finally the Chronic Stress Visualizer (4) that analyzes and visualizes the incidence of chronic stress over a selected period of time.

### 4.2. Context-Aware Chronic Stress Calculation Algorithm

With the purpose of continuous and efficient acquisition of the current user stress state (within predefined discrete time periods), we have employed the Hidden Markov Models (HMMs) [25–27] for the implementation of the context-aware stress calculation engine. HMM is a temporal probabilistic model in which the state of the process is described by a discrete random variable. The possible values of the variable are the possible states of the world.

**Figure 5.**
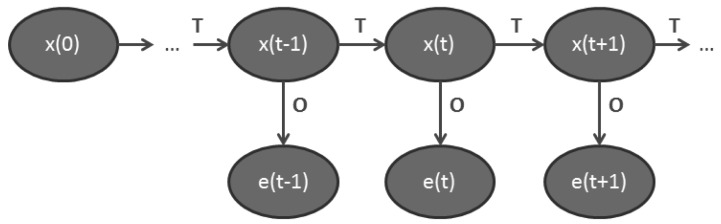
A general architecture of a first order Hidden Markov Model. Each oval shape represents a random variable. The random variable x(t) is the hidden state at time t with an initial state x(0). The random variable e(t) is the observation (evidence) at time t. The arrows in the diagram indicate conditional probabilities.

HMMs are used to represent non deterministic processes and consist of states, actions and observations (evidences). The observation of a state is determined by the conditional probability distribution of the state and is based on the Markov property, where the current state of the environment depends solely on the intermediate previous state and the associated action. In HMMs the state sequence is hidden and only the observations are visible. The transitions from one state to another matches probabilities that they will occur (Figure 5).

HMM is an ideal tool to integrate observations, while it is capable of addressing uncertainty in chronic stress modeling. HMM is defined by the:

- initial state distribution, *P*(*x*_0_=*i*),
- transition model *T* = {*t_ij_*} such that:
  (1) $$t_{\text{ij}}=P(X_{t}=j|X_{t-1}=i)$$
- nd observation model *O* = {*o_ij_*} such that:
  (2) $$o_{\text{ij}}=P(e_{t}|X_{t}=i)$$

To obtain current hidden state from observation we multiply the prior with the measurement probability using Bayes' rule:

(3) $$P(x_{i}=i|e_{t})=\alpha P(e_{t}|X_{t}=i)P(X_{t}=i)$$

In context-aware sensing the finite set of states represent different user-defined context profiles. The HMM chain models the transition processes through the states—this represents the user behavior during transitions from one context to another [28].

HMMs require a training procedure in order to later classify the activities. We have employed multiple observations supervised training [29]. The Viterbi algorithm is used to find the most likely state sequence that matches a given observation sequence given the HMM. To enable effective learning, we initialized HMM parameters in a supervised learning stage, in which pairs of observation sequence and state labels were used. Data labels are received by the mobile application questionnaire during a one week learning period. To construct the initial training example we took a set of last valid features around the timestamp of an individual's self-report. The same window size was used in the Viterbi algorithm during classification.

### 4.3. Collecting Stress Reports from the User

**Figure 6.**
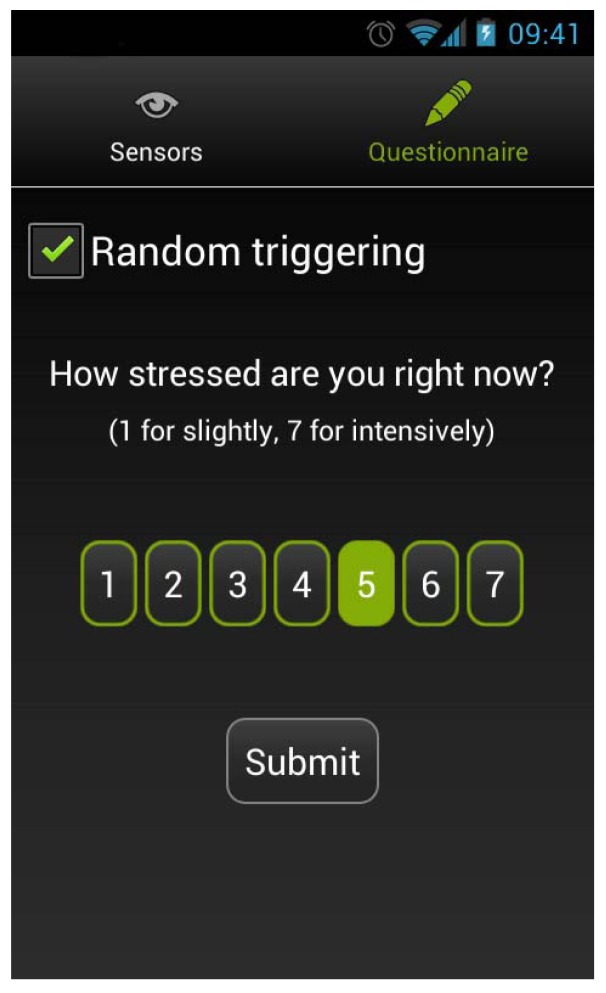
The mobile Android application was developed to deliver the chronic stress questionnaire. The 7-point Likert scale is visible in the center of screen.

In order to be able to train the presented model we implemented a simple questionnaire inside our mobile application (Figure 6). It is randomly triggered to sample current stress levels for seven consecutive days. The signaling time is selected for each block of 60 min with a requirement that signals should be triggered at least 15 min apart. Randomly triggered signals are not related to external events. That keeps our methodology independent.

The questionnaire consists of a single question, asking the user to estimate the current tension level from 1 (slight) to 7 (intense) on a 7-point Likert scale. Results are uploaded to the server's database where they are separated into a training set and a testing set in order to learn the initial HMM parameters.

The proposed supervised training is subjective to the user which can potentially lead to erroneous results, since user perception of current stress is not always optimal. The main reason why presented approach does not include training system objectively, e.g., by the experts in stress recognition (physician or psychologist), is that one of the initial aims was to design an autonomous service which can perform its tasks without external intervention. It is also not suitable to generalize the circumstances of stress incidences through entire population while they vary between individuals. This is why we were urged to make use of subjective model training.

A 7-point scale has been chosen based on some other well-known questionnaires like NASA Task Load Index (TLX) [30], which is a subjective workload assessment tool and can be used to assess workload in various human-machine environments. It consists of six scales, each presented as a line with a title and descriptors at each end (e.g., high/low). The evaluation of the proposed questionnaire has been made by measuring current stress level (using EDA sensor) in parallel (see Section 6–Clinical Evaluation).

### 4.4. Extracting Features

**Figure 7.**
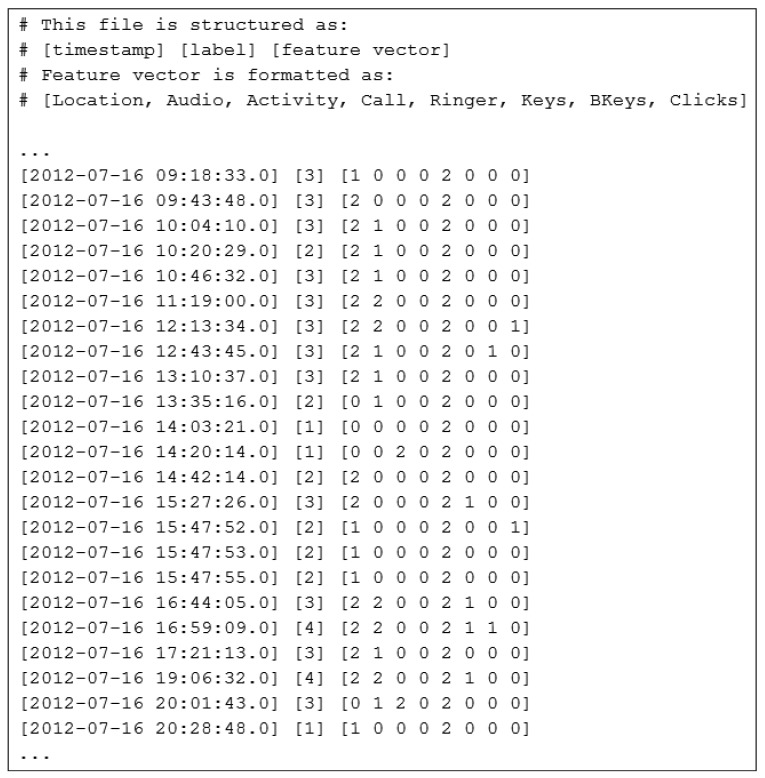
A part of generated training set with labeled feature vectors. Our model consists of seven states (discrete label values within interval [1–7]) and eight sensors per observation. Each observation sensor can take three values within the interval of [0–2].

Since event values are generated from different types of sensors (sensing location, activity and ambient), it makes it difficult for the context engine to process all types of values. Also, some of the values (e.g., ringer status) are given using non-technical context attributes that need to be adequately represented as numeric values. Consequently we transform data from database entries into discrete variables, which have values within the interval of [0, 2]. Each feature vector consists of eight sensor readings and is labeled with the corresponding value obtained by questionnaire (Figure 7).

### 4.5. Visualization of the Incidence of Chronic Stress

After the training period is completed, the system is able to recognize ongoing stress values and visualize the calculated context triplets (see Section 5-Results). It is important to realize that it does not require any further input by the user. Results can be presented by Stress Visualizer component and rendered inside the Internet browser in a user friendly manner. Visualization is achieved by using graphical web visualization tools such as Google Charts.

## 5. Results

We defined the HMM states by using a discrete variable named tension score with values from 1 to 7 (seven states). Tension score describes the state of a user's current tension, caused by external stressors (irritants). Each time the tension score is calculated using the HMM algorithm, the result is saved to the database together with current timestamp and the valid observation data (context triplet).

**Figure 8.**
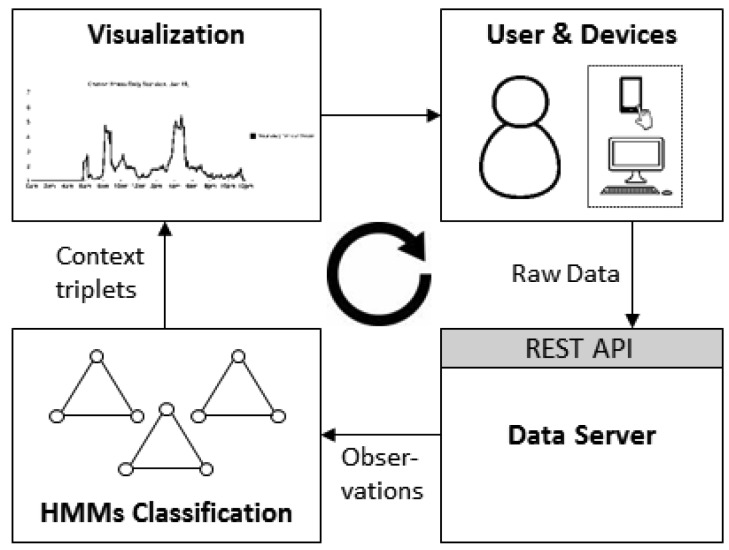
Contextual data (LOC, ACT and AMB) are provided by user devices. Preprocessed data (observations) is used to calculate tension score (HMMs classification), which is then visualized together with corresponding observations and timestamps (context triplet).

HMM based engine is realized on the basis of existing implementation of Hidden Markov Models (Jahmm [31]) using Java programming language. Additionally we have implemented supervised training part in order to obtain initial model parameters from our training data (Figure 8).

The current version of the Context-aware Chronic Stress Recognition System is undergoing testing within a group of test participants. The preliminary results are very encouraging and suggest some new possibilities of further research in the way of enhanced calculation models and stress assessment methodologies, with the goal of improving the training process. In the near future, we will extend testing to a broader population of individuals from various professions and demographic groups.

It is very important for the user to be able to access a visualization of the chronic stress occurrences, in order to help him/her recognize his/her ongoing stress situations. This represents the main added value of our work for the end users and may contribute to the commonly observed pattern of changing user behavior over time.

For example, if the user recognizes chronic stress situation such as a lack of sleep, he/she will be able to eliminate it (e.g., by going to bed one hour earlier) in order to minimize future health risks. Such a concept is well known among behavioral therapists. In this field, the assumption is that the act of recording everyday thoughts, feelings, and behaviors may help modifying user's undesired behavior.

**Figure 9.**
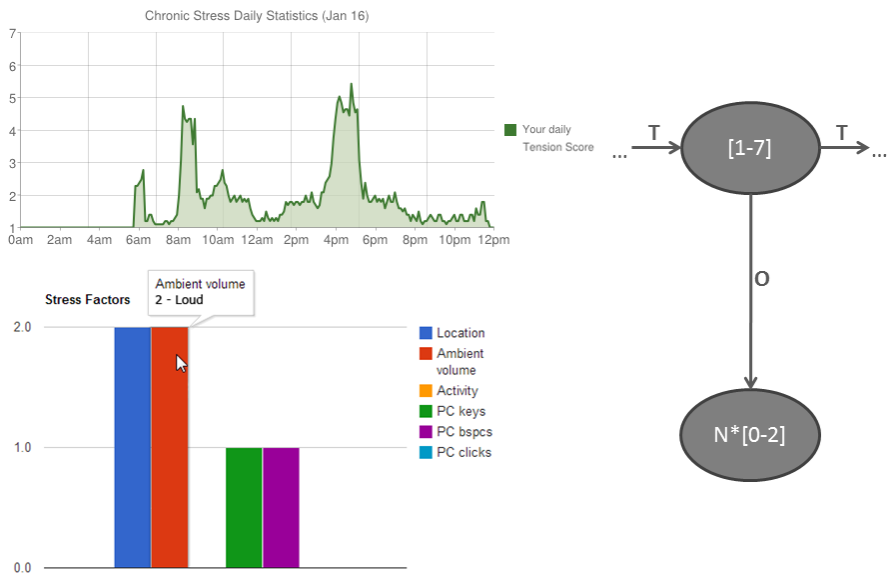
User context triplets are visualized inside a Web graphical user interface in a descriptive form. The upper chart shows daily tension score statistics (values from 1 to 7) based on HMM states. While it is clickable user is able to overview the corresponding observations for each plotted tension score (the bottom chart).

Currently all results obtained by the calculation engine are visualized and can be seen inside the visualization dashboard. Figure 9 shows an example of daily stress statistics for the selected user mapped to proposed calculation model.

The following section will provide clinical evaluation of our proposed supervised training part.

## 6. Clinical Evaluation

Clinical evaluation of the proposed subjective training methodology has been made using electrodermal activity (EDA) sensors. We measure EDA in order to compare clinical data to our data labels, which are crucial for proper model training.

EDA refers to electrical changes measured at the surface of the skin that arise when the skin receives innervating signals from the brain. It has been shown [32] that for most people, when experiencing emotional arousal, increased cognitive workload or physical activity, the brain send signals to the skin in order to increase the level of sweating. The preferred way of assessing EDA is by measuring of conductance rather than resistance. This is because of the nature of the skin which acts as a series of parallel resistors. Therefore, the most common unit of measurement in EDA is the microsiemen (μS).

**Figure 10.**
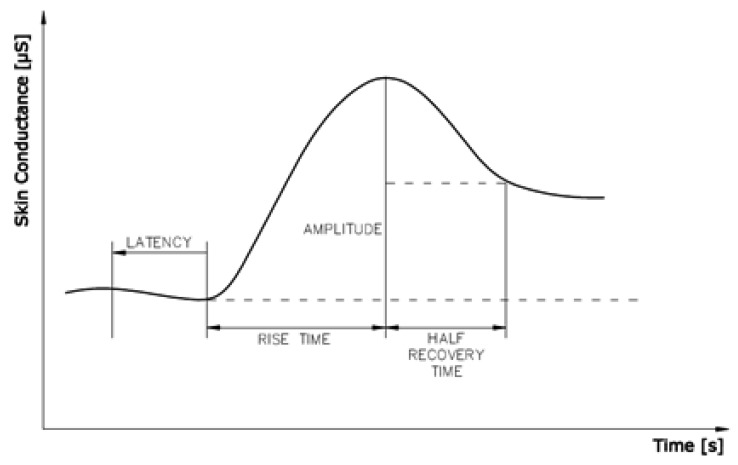
SCR's appear after breaking a sweat. *Latency*—interval between stimulus onset and SCR initiation; *Amplitude*—phasic increase in conductance following stimulus onset; *Rise time*—interval between SCR initiation and SCR peak; *Half recovery time*—interval between SCR peak and point of 50% recovery of SCR amplitude.

Skin conductance measurement is characterized into two types—tonic and phasic. Tonic skin conductance is referred to as Skin Conductance Level (SCL) and it slightly varies over time. Tonic changes typically occur in a period of minutes. Phasic skin conductance measurements are most often associated with short-term events. They occur in the presence of discrete environmental stimuli such as noise, smell, fear, anticipation, *etc.* Phasic changes usually show up as exponential increases in the skin conductance. Such change is referred to as Skin Conductance Response (SCR). Based on the frequency distributions of response latencies to a stimulus it is common to use 1.5 to 6.5 seconds latency window. Any SCR that begins inside this time window following stimulus onset is considered to be induced by that stimulus. A SCR shows a rapid incline to the peak and a slow decline to the baseline (Figure 10) [33].

**Figure 11.**
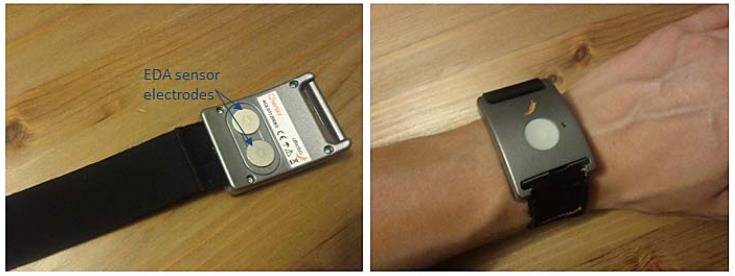
Wearable wristband EDA sensor.

We measure EDA (conductance amplitude) by employing wearable, sleek-form wristband sensor [34] that allows us to conduct long-term measurements. The sensor has an embedded battery (24-hour battery life when logging), internal storage for three months of data and a Bluetooth wireless connectivity allowing us to monitor data in real time (Figure 11).

**Figure 12.**
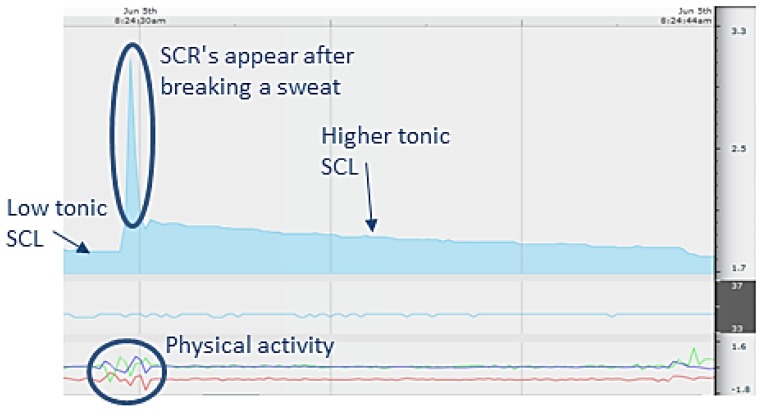
EDA sensor has embedded 3D accelerometer and thermometer allowing us to interpret the measurement results correctly.

The signals acquired by EDA sensors are often corrupted by the users' movements during the measurement which is called motion artifact. Results can also be distorted by the users' physical activity or high environmental temperature. In order to avoid such misleading measurements EDA sensor embeds 3D accelerometer and thermometer. Thus it is easy to detect high conductance levels when they are triggered by user's physical activity, wrist movements (motion artifact) or raised environmental temperature (Figure 12).

Average tonic level of an individual during rest condition and in the absence of any external stimulus is called baseline (*SCL*_min_). The best way to get a baseline is to perform long-term measurements and look for the lowest smooth period of skin conductance where there is no physical movement and where the temperature equals to body temperature. According to Csikszentmihalyi and Larson [6] seven consecutive days should be enough to sample user's everyday life. In a similar way we obtain highest smooth period of skin conductance which is marked as *SCL*_max_ The individual's present SCL can then be expressed as a proportion according to the following equation:

(4) $${\text{SCL}}_{\text{rel}}=\frac{\text{SCL}-{\text{SCL}}_{\min}}{{\text{SCL}}_{\max}-{\text{SCL}}_{\min}}$$

Similarly the individual's present SCR can also be expressed as a proportion, where *SCR*_min_ can be assumed to be zero and *SCR*_max_ is recognized as a measured max response to discrete event:

(5) $${\text{SCR}}_{\text{rel}}=\frac{\text{SCR}}{{\text{SCR}}_{\max}}$$

**Figure 13.**
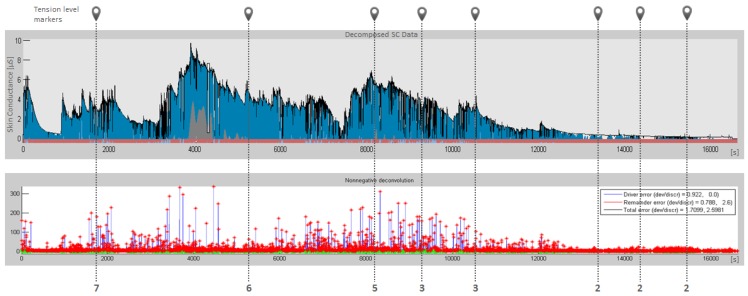
Sequence of the decomposition of skin conductance data by means of nonnegative deconvolution (**top**). Tonic SC activity (grey) is separated from phasic SC data (blue). High-pass filtered EDA signal is shown (**bottom**). The red stars indicate the automatically detected SCR peaks. Tension level markers (vertical lines) show user's subjective estimation of his current stress condition answering questionnaire.

In the studies performed by Setz [35], analysis of the EDA data showed that the distributions of the EDA peak height and the SCR peak rate carry information about the stress level of a person. To decompose skin conductance data nonnegative deconvolution is applied as described by Benedek [36]. Training data labels, obtained by questionnaire, are then compared to EDA readings (Figure 13).

After a detailed review of the decomposed and filtered skin conductance data we can evaluate the user's ability to estimate his current stress by using proposed questionnaire. From the observed combination of signal peaks height and rate, we can notice, that an average user is able to make a good assessment, but even if the user constantly underestimates or overestimates his exposure to stress, we can (due to our long-term measurements) still identify situations which stand out from the long-term average. Additionally, while HMM is a statistical model, some potential reports of one-time events (related to acute stressors) do not affect the final calculation.

## 7. Conclusions and Future Work

To the best of our knowledge, the system presented herein is the first that enables one to recognize chronic stress situations on the basis of user's context. By training our HMM model, using combination of well-established methods of stress measurement (signal-contingent recording) and supervised learning algorithm, we have provided a novel methodology to evaluate chronic stress. The visualization module was implemented to graphically emphasize the most important stress situations with high repetition frequency. The main added value of our research work is achieved by enabling end users to successfully recognize and affect her/his ongoing stressors in order to minimize future health risks.

The presented research is concerned with the usage of a first-order HMM for reasoning about user context in order to determine the presence of a chronic stress. Even though the current solution already satisfies the requirements of the methodology, a look beyond it reveals a rich variety of possible improvements. Some of them include the enhancements of the HMM algorithm with the goal of performance and accuracy improvements. A number of interesting algorithms exist in the literature, for example the Layered HMM (LHMM) [37] which can facilitate the learning procedure. We are also considering the usage of the particle filters algorithm. The latter is used in various fields of artificial intelligence and robotics and allows for modeling of continuous state spaces very effectively [38]. In addition we will conduct extensive testing of the proposed methodology with the emphasis on usage in the real life scenarios.

## Figures and Tables

**Table 1.** Employed context information probes with data description and possible data values.

**Probe**	**Descriptions**	**Context Mapping**	**Data Values**
Location	User's mobile location	LOC	0–unknown
			1–home
			2–at work
Ambient volume	Ambient audio features	AMB	0–silent
			1–low volume
			2–high volume
Physical activity	User's physical activity	ACT	0–not active
			1–low activity
			2–high activity
Call status	User's current mobile phone call status	ACT	0–idle
			1–ringing
			2–in a call
Ringer status	User's current mobile phone ringer status	ACT	0–silent
			1–vibrator
			2–normal
PC keys	PC keys pressed	ACT	0–not active
			1–low activity
			2–high activity
PC backspace keys	PC backspace keys pressed	ACT	0–not active
			1–low activity
			2–high activity
PC clicks	PC mouse clicks	ACT	0–not active
			1–low activity
			2–high activity
